# Digital trends in autism: a scoping review exploring coverage of autism across YouTube, Twitter, and Facebook

**DOI:** 10.3389/fdgth.2023.1222187

**Published:** 2023-09-27

**Authors:** Aysha Jawed, Heather Graham, Jennifer Smith

**Affiliations:** ^1^ Charlotte R. Bloomberg Children's Center, Baltimore, MD, United States; ^2^ Department of Pediatric Social Work, Charlotte R. Bloomberg Children's Center, Baltimore, MD, United States; ^3^ Department of Pediatric Nursing, Charlotte R. Bloomberg Children's Center, Baltimore, MD, United States; ^4^ Infant Neurodevelopment Center, Kennedy Krieger Institute, Baltimore, MD, United States; ^5^ Johns Hopkins School of Nursing, Johns Hopkins University, Baltimore, MD, United States

**Keywords:** autism, neurobehavioral disorder, neurodiversity, social media, developmental disability

## Abstract

Autism continues to be a leading neurodevelopmental disorder across adult and pediatric populations that transcends racial, ethnic, age, and socioeconomic groups worldwide. Autism care and treatment also exerts immense costs on the healthcare system and lost productivity which are partly attributed to the existing resource limitations globally. Organizations, campaigns, and policies exist worldwide in increasing equity and accessibility of resources and services to individuals with autism. In the context of our digital era, a wealth of information is also more readily available on autism through electronic communication including social media platforms. As YouTube, Twitter and Facebook are ever-growing and among the leading social media platforms in contemporary times, examination of content covered on autism across these communication mediums is timely and warranted. This review consolidates findings from 32 sources on the sources, formats, and nature of content covered on YouTube, Twitter, and Facebook pertaining to a wealth of dimensions surrounding autism. Strengths and limitations of the studies and endeavors are presented. Implications for future campaign development, health equity, health policy, neurodiversity, and patient care are also delineated. Lastly, recommendations for future research and practice are discussed which present directions for tapping into the potential of YouTube, Twitter, and Facebook as health communication mediums across the ever-changing autism landscape.

## 1. Introduction

Autism is a neurobehavioral disorder and development disability that is prevalent across the world among all racial, ethnic, age, and socioeconomic groups (1). Oftentimes, individuals with autism will experience challenges with their communication and social interactions as well as increased repetitive behaviors, restricted interests, and unusual responses to sensory input (1). The trajectory of autism is chronic and lifelong in nature. Children are often diagnosed with autism before the age of 5 years and can live with autism for a very long time (2). Autism is more prevalent among boys than girls (3). There are three severity levels based on level of supports in autism across the spectrum which are detailed in the Diagnostic and Statistical Manual of Mental Disorders (DSM-V) (4). It is also crucial to note that autism is a distinct neurotype which may have disabling effects due to insufficient accommodation of sensory needs. In addition, autism is on a spectrum from mild to severe which may not always encompass the specific classification categories for severity level of autism detailed in the DSM-V. However, autistic individuals are also able to fully participate in all aspects of society with appropriate support based on severity level.

As more organizations involved in disseminating knowledge and resources on autism have extended their presence into online spaces (e.g., the American Academy of Pediatrics, CDC, Autism Speaks, March of Dimes), examining these electronic communications could help identify content that could be prioritized in future mobilization of resources and advocacy efforts in heightening awareness and support of autism research and practice. Further, as several of these organizations have extended beyond traditional forms of media onto nontraditional forms (e.g., social media), critical examination of the wealth of disseminated information combined with content shared by a range of primary and lay sources is warranted.

In this digital era, social media is rising as a leading communication medium for a wealth of topics including health-related ones. Oftentimes content covered on social media is reflective of the existing state of trends across communities, countries, and the world. As increasingly more people are living longer with autism, examining the content shared on social media among older and newer generations can help uncover the value of content that is helpful or not helpful for autistic individuals and their networks. YouTube, Twitter, and Facebook are among the most popular social media platforms in our current times (5). In addition, these social media platforms include content delivered in a wealth of formats that can reach individuals across varying levels of literacy.

Notably, there were no published studies on autism-related across TikTok, Reddit, Instagram, and other social media. However, the most prevalent, widely used social media platforms (YouTube, Facebook, and Twitter) continue to be minefields of knowledge transfer and dissemination on autism as well as risk factors for heightening misinformation and disinformation. Several content analyses have examined the most widely viewed videos on autism as well as posts, communication across communities of individuals with autism and their networks, and pictures.

To date, there has not been a single review that has consolidated the wealth of existing knowledge, research and practice across published studies and lay sources on the coverage of autism across the most popular social media platforms. As the global reach of social media continues to rise in our digital era, undertaking an exploration to understand a wealth of dimensions surrounding autism across these social media platforms is timely. This review will present the key findings from published studies on autism-related content covered on social media. These findings include more descriptive information about autism along with a range of considerations pertaining to activities of daily living, achieving health equity, challenges and uncertainties, caregiver impact, navigation of healthcare, educational, and vocational systems, among many more. It follows that the constellation of these key findings will elucidate a more holistic and biopsychosocial picture on the diversity of complexities surrounding autism across all developmental stages.

The goals of this review are the following: (1) present the constellation of sources and formats among the electronic communication pertaining to autism across YouTube, Twitter, and Facebook; (2) synthesize the existing understanding and coverage of content across a range of determinants and dimensions on autism across these social media platforms; and (3) delineate recommendations for future research and practice that can draw on the strengths and build off the limitations of social media as a communication medium in autism awareness, care, and treatment.

## 2. Materials and methods

### 2.1. Search strategy

Following the PRISMA guidelines for a scoping review, an extensive search of the academic literature on autism coverage across the social media platforms YouTube, Twitter, and Facebook were conducted in July 2023. The medical, public health, educational, and psychosocial databases reviewed were the following: Medline (Northfield, IL, USA), APA PsychInfo, Cochrane Review, Academic Search Premier, CINAHL, ERIC and EBSCO. Key terms used across searches were the following: autism, autistic, Asperger's, social media, YouTube, Twitter, and Facebook.

### 2.2. Eligibility criteria

Only published studies on coverage of autism across prominent social media platforms were included in this review. Given the nature of this review, secondary and tertiary sources from magazines, newspapers, blogs, posts and other communication mediums were excluded. Any sources that did not share content on autism were ultimately excluded.

### 2.3. Procedure

Published studies constituted the units of analysis for this review. These published studies were primary sources from primarily descriptive, observational studies conducted on critically examining autism-related content across the top widely prevalent social media platforms. Content on autism covered across these published studies involved determinants, dimensions, and domains surrounding autism inclusive of chronic care management, global development, educational and vocational opportunities, neurodiversity, health equity and inclusivity among more content considerations.

All of the authors independently reviewed all titles and abstracts that populated across each database. Differences concerning full-text inclusion and coverage of autism were resolved through consensus. All of the authors then independently abstracted data across all included studies on sources, formats, nature of content, and any additional descriptive information on the kinds of videos explored in each source. Results were subsequently compared and any discrepancies were fully resolved through active discussions.

## 3. Results

A cumulative total of 315 records were identified across the databases reviewed from the past 50 years. One hundred forty six of these records were duplicates and subsequently excluded. Among the remaining 169 records, 122 of them were excluded for either missing full-text, referenced a different target population, and goals of research did not assess coverage of dimensions surrounding autism. Next, 47 full-text articles were thoroughly examined for inclusion in this scoping review.

Among them, 15 were further excluded for one or more of the following reasons: (1) presented different social media platforms across content covered; (2) missing originality of work; (3) the target population did not involve individuals with autism; (4) there were different topics covered which ultimately changed the focus in the sources; and (5) the goals of several published studies were divergent.

**Figure 1:**
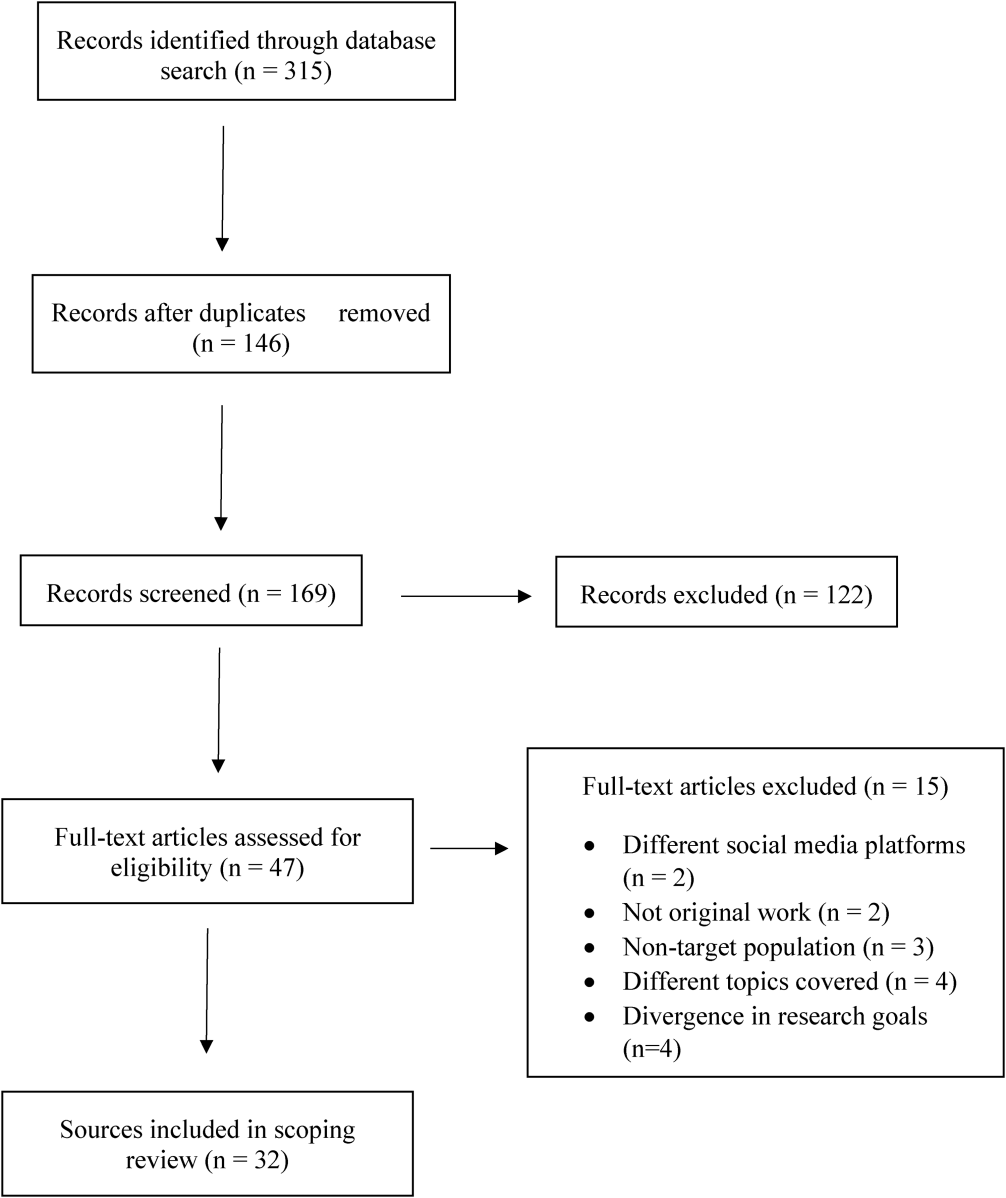
Literature review flowchart.

Thirty two articles ultimately met the criteria for coverage of autism across YouTube, Twitter, and Facebook as delineated in Figure 1 (6–37). Majority of the studies involved conducting cross-sectional and descriptive content analyses. A wide range of content was covered across studies including treatments and therapies, community outreach and engagement for individuals with autism and their networks, narratives on navigating everyday challenges for children with autism, inclusivity considerations, and much more. All content across the literature included in this review were in English. A comprehensive breakdown of the sources and formats of autism content covered across YouTube, Twitter, and Facebook from the published literature can be found in Table 1. Further, autism content covered across each of these social media platforms are delineated in Table 2.

**Table 1:** Sources and formats of autism content covered across YouTube, Twitter, and Facebook.

	YouTube	Twitter	Facebook
Sources	Mothers of autistic children Freitas and Gaudenzi (14); family members Fusaro et al. (10); parents Lloyd et al. (13); professionals Lloyd et al. (13); professional, consumer, television, and internet sources Bellon-Harn et al. (8); individuals with autism Angulo-Jimenez and DeThorne (6); professional societies, educational centers, TV programs and parents of children with autism Azer et al. (12); primarily non-professionals Kollia et al. (7); children with autism, their caregivers and individuals in their family, social, and employer-based networks Bakombo et al. (35); physicians and psychologists Cortes Cavalcante et al. (36)	Organizations, news networks, celebrities, and consumers Deriss (20); organizational sources included commercial/for-profit, non-profit, clinical, academic and other organizations Bellon-Harn and Manchaiah (8); individuals with autism and their significant others Bellon-Harn and Manchaiah (8); consumers Karusala et al. (23); autism bloggers which included consumers (individuals with autism and their caregivers) and professional organizations Saha and Agarwal (25); bloggers provided by Autism Speaks Saha and Agarwal (27); users from organizations, caregivers of children with autism, companies, charities for research on autism, and individuals with autism Corti et al. (18); Autism Speaks and caregivers of children with autism; special education, rehabilitation centers, child and adolescent psychiatrists, and municipalities Goksel et al. (37)	Autism-based organizations Bail (32); patients with autism, their caregivers, researchers, and healthcare providers Zhao et al. (30); primarily caregivers followed by teachers in special education, suppliers of ASD-related products and services, healthcare providers, academic researchers, as well as other individuals involved in the direct or indirect care of children with autism Mohd Roffeei et al. (31); caregivers of children with autism Antunes and Dhoest (34)
Formats	Testimonials of mothers with autistic children Freitas & Gaudenzi (14); images and texts elucidating the learning evolution of autistic children Freitas & Gaudenzi (14); five slide-show style videos with photos and texts Lloyd et al. (13); testimonials (narratives) posted by parents of children with autism Azer et al. (12); home videos Fusaro et al. (10); interviews Lloyd et al. (13); commentaries presented by parents across images Lloyd et al. (3); personal videos and television show clips Kollia et al. (7); narratives posted by children with autism, their caregivers and individuals in their family, social, and employer-based networks Bakombo et al. (35)	Hashtags	Audiovisual, written messages, comments

**Table 2:** Autism content covered across YouTube, Twitter, and Facebook.

	YouTube	Twitter	Facebook
I. Navigating everyday life			
Daily lived experiences	Freitas and Gaudenzi (14), Lloyd et al. (13), Azer et al. (12), Bakombo et al. (35)	Sahaand Agarwal (25), Goksel et al. (37)	Zhao et al. (30), Antunes and Dhoest (34)
Daily challenges	Azer et al. (12)	Saha and Agarwal (26), Saha and Agarwal (27), Gauld et al. (17), Goksel et al. (37)	Zhao et al. (30), Mohd Roffeei et al. (31), Antunes and Dhoest (34)
Hygiene and cleanliness		Deriss (20)	
Impact of the pandemic on daily life		Corti et al. (18)	
Raising children with autism		Saha and Agarwal (26)	Mohd Roffeei et al. (31)
Employment		Sani-Bozkurt (19)	
II. Stressors and triggers			
Grief and loss	Freitas and Gaudenzi (14), Lloyd et al. (13)		Mohd Roffeei et al. (31)
Psychological distress	Lloyd et al. (13)		
guilt	Lloyd et al. (13)		
Financial struggles	Lloyd et al. (13), Kollia et al. (7)		
Caregiver relationship strain	Lloyd et al. (13)		
Social isolation	Freitas and Gaudenzi (14), Lloyd et al. (13)		Antunes and Dhoest (34)
Electromagnetic field pollution			Zhao et al. (30)
III. Conceptualization and descriptive epidemiology of autism			
Definition of autism		Sani-Bozkurt (19), Goksel et al. (37)	
Facts and statistics	Lloyd et al. (13)	Goksel et al. (37)	
Representation of the color blue	Freitas and Gaudenzi (14)		
Self-identification of autism	Angulo-Jimenez and DeThorne (6)		
Features and characteristics of autism (e.g. eye contact patterns, speech, social communication, cognitive skills)	Lloyd et al. (13), Bakombo et al. (35), Cortes Cavalcante et al. (36)	Sani-Bozkurt (19)	Mohd Roffeei et al. (31), Zhao et al. (30)
Signs and symptoms of autism	Lloyd et al. (13), Bellon-Harn et al. (9), Bakombo et al. (35)	Bellon-Harn et al. (8)	
Demystifying misinformation		Bellon-Harn et al. (8)	
IV. Behaviors			
Repetitive behaviors	Kollia et al. (7)		
Screaming and yelling	Kollia et al. (7), Bakombo et al. (35)		
Self-injurious behaviors	Kollia et al. (7), Bakombo et al. (35)		
Playing with toys	Kollia et al. (7)		
Self-stimulatory behaviors (e.g. hand flapping, grunting, excessive blinking)	Bakombo et al. (35)		
Crying	Bakombo et al. (35)		
V. Recommendations for therapies and treatment			
Behavioral management strategies	Azer et al. (12), Freitas and Gaudenzi (14)	Bellon-Harn et al. (8)	Zhao et al. (30), Mohd Roffeei et al. (31)
Developmental therapies	Freitas and Gaudenzi (14)		
SUS model for treatment	Freitas and Gaudenzi (14)		
Mixed services partly financed by government	Freitas and Gaudenzi (14)		Antunes and Dhoest (34)
Private treatments	Freitaz and Gaudenzi (14)		
Speech language therapy	Lloyd et al. (13), Kollia et al. (7)		Abel et al. (33)
Applied behavior analysis	Kollia et al. (7)	Bellon-Harn et al. (8)	Abel et al. (33)
Psychological interventions	Kollia et al. (7)		
Complementary alternative practices (e.g. animal, art, herbal therapies, cannabis)	Kollia et al. (7)	Bellon-Harn et al. (8)	Abel et al. (33)
Additional sources of information on autism (e.g. websites)	Kollia et al. (7)	Deriss (20)	
Curing autism	Lloyd et al. (13), Angulo-Jimenez and DeThorne (6), Lacruz-Perez et al. (11)	Deriss (20)	
Medication selection and administration (including vitamins and supplements)	Freitas and Gaudenzi (14), Lloyd et al. (13)		Mohd Roffeei et al. (31), Antunes and Dhoest (35)
Diets	Azer et al. (12), Kollia et al. (7)		Mohd Roffeei et al. (31)
Utilization of google glasses		Bellon-Harn et al. (8)	
Toilet training			Mohd-Roffeei et al. (31)
Play therapy			Zhao et al. (30)
Multidisciplinary treatment	Cortes Cavalcante et al. (36)		
VI. Inequities			
Inaccessibility of schools	Lloyd et al. (13)		
Prejudices by the school system	Freitas and Gaudenzi (14)		Antunes and Dhoest (34)
Prejudices by the community	Freitas and Gaudenzi (14)		Antunes and Dhoest (34)
Limitations in accommodations	Angulo-Jimenez and DeThorne (6)		
Civil Issues (e.g. criminal justice, policing)		Bellon-Harn et al. (8)	
Call for improving social support in educational and vocational contexts		Karusala et al. (23)	
Inclusivity		Sani-Bozkurt (19)	Antunes and Dhoest (34)
Social acceptance		Sani-Bozkurt (19)	
VII. Education			
Inclusive and equitable educational opportunities in specialized schools	Lacruz-Perez et al. (11), Kollia et al. (7)	Goksel et al. (37)	Antunes and Dhoest (34)
Teaching autistic children how to write		Saha and Agarwal (26)	
Behavioral and social aspects of going to school		Beykikhoshk et al. (28)	
Language		Beykikhoshk et al. (28)	
Intelligence		Beykikhoshk et al. (28)	
Reading			Mohd Roffeei et al. (31)
School subjects			Mohd Roffeei et al. (31)
Communication skills			Mohd Roffeei et al. (31)
VIII. Support for individuals with autism and their caregivers			
Coping strategies for caregivers	Lloyd et al. (13)	Saha and Agarwal (26), Saha and Agarwal (27)	
Resources for child and family support	Kollia et al. (7)		
Support groups	Kollia et al. (7)		Zhao et al. (30), Mohd Roffeei et al. (31), Abel et al. (33)
Sense of community	Freitas and Gaudenzi (14), Lloyd et al. (13), Azer et al. (12)	Corti et al. (18)	
Well-being		Corti et al. (18)	Ward et al. (29)
Financial resources (e.g. supplemental security income)			Zhao et al. (30), Abel et al. (33)
Celebrating achievements and milestones			Zhao et al. (30), Mohd Roffeei et al. (31), Antunes and Dhoest (34)
Prayers			Mohd Roffeei et al. (31), Abel et al. (33)
Situational appraisal			Mohd Roffeei et al. (31)
Meet-ups			Abel et al. (33)
IX. Developmental considerations			
Children	Freitas and Gaudenzi (14), Fusaro et al. (10), Angulo-Jimenez and DeThorne (6), Azer et al. (12), Kollia et al. (7)		
Adults	Angulo-Jimenez & DeThorne (6), Kollia et al. (7)		
Atypical development of children in meeting developmental milestones	Freitas and Gaudenzi (14)		
Communication impairment	Freitas and Gaudenzi (14), Bakombo et al. (35)		
X. Neurodiversity			
Individual differences and uniqueness	Freitas and Gaudenzi (14), Kollia et al. (7), Bakombo et al. (35)	Saha and Agarwal (26), Sani-Bozkurt (19)	Antunes and Dhoest (34)
Psychiatric co-morbidities	Angulo-Jimenez and DeThorne (6)		Antunes and Dhoest (34)
Co-morbid sensory complications	Angulo-Jimenez and DeThorne (6), Kollia et al. (7)		
Autism as an inherent part of identity	Kollia et al. (7)		
Empowerment in embracing neurodiversity		Bellon-Harn et al. (8)	
Asset and superpower		Skafle et al. (15)	
XI. Medical model			
Autism as a disorder or defect	Angulo-Jimenez and DeThorne (6)		
XII. Autism awareness and advocacy			
World autism awareness day		Ahmed et al. (21), Karusala et al. (23), Sani-Bozkurt (19)	
Autism speaks advocacy organization		Beykikhoshk et al. (22), Karusala et al. (23)	
Action summit on autism		Beykikhoshk et al. (22)	
Representation of the color blue		Karusala et al. (23), Sani-Bozkurt (19)	
Books		Bellon-Harn et al. (8)	Abel et al. (33)
Media coverage		Bellon-Harn et al. (8)	
Celebrity endorsements		Bellon-Harn et al. (8)	
Fundraising		Bellon-Harn et al. (8)	Abel et al. (33)
Healthcare providers participating in interviews		Sani-Bozkurt (19)	
Community engagement		Karusala et al. (23)	Zhao et al. (30), Mohd Roffeei et al. (31)
Autism-related research	Bakombo et al. (35)		Zhao et al. (30), Mohd Roffeei et al. (31)
Jewelry			Abel et al. (33)
Trainings, workshops, and conferences			Zhao et al. (30)
Tattoos			Abel et al. (33)
XIII. Recreation			
Toys and gifts for children with autism	Azer et al. (12)		
Sports and recreational activities		Deriss (20)	
Play groups			Abel et al. (33)
XIV. Misinformation			
MMR vaccine	Azer et al. (12)		
Scarcity of vitamins	Azer et al. (12)		
Immunizations/vaccinations	Azer et al. (12), Kollia et al. (7)	Bellon-Harn et al. (8)	
Environmental pollution	Azer et al. (12)		
Toxicity	Azer et al. (12)		
Scarcity of amino acids	Azer et al. (12)		
Gluten-free diet as cure for autism	Azer et al. (12)		Antunes and Dhoest (34)
Asymmetrical brain injuries		Gabarron et al. (16)	

Among published studies examining autism-related content on YouTube, sources of content included the following: mothers of autistic children (14), family members (10, 35), parents (12, 13, 35), professionals (13), professional, consumer, television, and internet sources (8, 9), individuals with autism (6), professional societies, educational centers, TV programs (12), primarily non-professionals (7); children with autism, individuals in their social and employer-based networks (35); physicians and psychologists (36).

Across published studies on Twitter, content was posted by a diversity of sources that included organizations, news networks, celebrities, consumers (20) organizational sources that included commercial/for-profit, non-profit, clinical, academic and other organizations (8), individuals with autism and their significant others (8), consumers (23), autism bloggers which also included consumers (individuals with autism and their caregivers) and professional organizations (25), bloggers provided by Autism Speaks (27), users from organizations, caregivers of children with autism, companies, charities for research on autism, individuals with autism (18, 19), special education, rehabilitation centers, child and adolescent psychiatrists, and municipalities (37).

Sources posting autism-related content on Facebook included Autism-based organizations (32), patients with autism, their caregivers, researchers, and healthcare providers (30), primarily caregivers followed by teachers in special education, suppliers of Autism Spectrum Disorder (ASD) related products and services, healthcare providers, academic researchers, as well as other individuals involved in the direct or indirect care of children with autism (31), and caregivers of children with autism (34).

Among content presented in published studies on autism coverage across YouTube, formats of videos included testimonials of mothers with autistic children (14), images and texts elucidating the learning evolution of autistic children (14), five slide-show style videos with photos and texts (13), testimonials (narratives) posted by parents of children with autism (12), home videos (10), interviews (13), commentaries presented by parents across images (13), personal videos and television show clips (7), and narratives posted by children with autism, their caregivers and individuals in their family, social, and employer-based networks (35). All published studies of content and sentiment analyses on autism coverage across Twitter featured hashtags as the engagement metric. Lastly, all published studies on autism coverage across Facebook analyzed content in the form of audiovisual and written messages, oftentimes in the form of comments.

Findings were ultimately grouped into 14 content categories which were the following: (1) Navigating Everyday Life; (2) Stressors and Triggers; (3) Conceptualization and Descriptive Epidemiology of Autism; (4) Behaviors; (5) Recommendations for Therapies and Treatments; (6) Inequities; (7) Education; (8) Support for Individuals with Autism and Their Caregivers; (9) Developmental Considerations; (10) Neurodiversity; (11) Medical Model; (12) Autism Awareness and Advocacy; (13) Recreation; and (14) Misinformation. These categories were created by the authors based on the content covered across the published studies. The authors reviewed content from each study and resolved any discrepancies through active discussion to collectively propose categories that represented the wide range of content in these studies.

### 3.1. Navigating everyday life

There were several considerations covered in regard to navigating everyday life among autistic individuals and their networks. Daily lived experiences were accounted for in content reviewed across eight studies (12–14, 25, 30, 34, 35, 37). Eight studies also examined content on daily challenges (12, 17, 26, 27, 30, 31, 34, 37). Two studies uncovered content on raising children with autism (26, 31). Content on hygiene and cleanliness was reviewed in one study (20). Impact of the pandemic on daily life was explored in another study (18). Employment was assessed in one study (19).

### 3.2. Stressors and triggers

Several stressors and triggers were covered in published studies on autism content across social media. Grief and loss were accounted for in three studies (13, 14, 31). Psychological distress, guilt, and caregiver relationship strain were covered in one study (13). Financial struggles were assessed in two studies (7, 13). Social isolation was reviewed in three studies (13, 14, 34). Electromagnetic field pollution was accounted for in one study (30).

### 3.3. Conceptualization and descriptive epidemiology of autism

Multiple studies presented a diversity of content with respect to the conceptualization and descriptive epidemiology of autism. Two studies presented content on defining autism (19, 37). Facts and statistics on the descriptive epidemiology were uncovered in two studies (13, 37). Representation of the color blue as a visible indicator of autism awareness, inclusivity, and acceptance was depicted in the content reviewed in one study (14). Self-identification of autism was examined in one study (6). Features and characteristics of autism (e.g., repetitive behaviors, self-stimulatory behaviors, communication impairments) were covered in six studies (13, 19, 30, 31, 35, 36). Signs and symptoms of autism for diagnostic consideration with respect to behaviors, communication, and development were depicted in four studies (8, 9, 13, 35). One study yielded content on demystifying misinformation (8).

### 3.4. Behaviors

Several behaviors were also reviewed among published studies on autism-related content across social media. Repetitive behaviors were covered in one study (7). Screaming, yelling, and self-injurious behaviors were reviewed in two studies (7, 35). Self-stimulatory behaviors (e.g., hand flapping, grunting, excessive blinking) was covered in one study (35). Crying was explored in another study (35). Playing with toys was accounted for in a different study (7).

### 3.5. Recommendations for therapies and treatment

A multitude of recommendations for therapies and treatment on autism were covered across published studies examining autism-related content across the prominent social media platforms. Five studies yielded content on behavioral management strategies (8, 12, 14, 30, 31). Developmental therapies, SUS model for treatment and private treatments were covered in one study (14). Mixed services partly financed by the government were explored in two studies (14, 34). Three studies examined content on speech language therapy (7, 13, 33). Three studies accounted for content on Applied Behavior Analysis (7, 8, 33). One study presented content on psychological interventions (7). Three studies covered content on complementary alternative practices (7, 8, 33). Additional sources of information were accounted for in two studies (7, 20). Four studies presented content on curative thoughts and approaches related to autism (6, 11, 13, 20). Four studies presented content on medication selection and administration (13, 14, 31, 34). Diets were covered in three studies (7, 12, 31). Utilization of Google glasses were reviewed in one study (8). Toilet training was explored in one study (31). Play therapy was explored in one study (30). Lastly, multidisciplinary treatment was featured in one study (36).

### 3.6. Inequities

There was also coverage of inequities across studies in this review on social media content related to autism. One study presented content on inaccessibility of schools for children with autism. Two studies described content on prejudices endured by the school system as well as the community (14, 34). One study covered content on limitations in accommodations (9). One study yielded content on civil issues (8). Another study explored content on improving social support in educational and vocational contexts (23). Inclusivity was covered in two studies (22, 37). Lastly, social acceptance was accounted for in one study (19).

### 3.7. Education

A multitude of considerations surrounding education for autistic individuals was reviewed in published studies on autism-related content across social media. Four studies presented content on inclusive and equitable educational opportunities in specialized schools (7, 11, 34, 37). One study covered content on teaching autistic children how to write (26). Another study examined content on behavioral and social aspects of going to school, language, and intelligence (28). One study explored content on reading, school subjects and communication skills (31).

### 3.8. Support for individuals with autism and their caregivers

Several factors for varying forms of support were also accounted for in published studies on autism-related content across social media. Coping strategies for caregivers were covered in three studies (13, 26, 27). One study included content on resources for child and family support (7). Four studies featured content on support groups (7, 30, 31, 33). Four studies explored content on creating and sustaining a sense of community (12–14, 18). Well-being was covered in two studies (18, 29). Financial resources were included in two studies (30, 33). Three studies presented content on celebrating achievements and milestones (30, 31, 34). Two studies reviewed content on prayers (31, 33). One study accounted for content on situational appraisal (31). Lastly, one study featured content on meet-ups (33).

### 3.9. Developmental considerations

Developmental considerations were also a focus of social media content on autism reviewed across published studies. Five studies examined content pertaining to children (6, 7, 10, 12–14). Two studies covered content related to adults (6, 7). One study included content on atypical development of children in meeting developmental milestones (14). Two studies presented content on communication impairment (14, 35).

### 3.10. Neurodiversity

Neurodiversity encompasses the unique individuality of our global population across a wide range of features and characteristics that form our population. Neurodiversity extends beyond the neurobehavioral context and includes differences across developmental, psychological, cognitive, physiological, and socioemotional domains. The concept of neurodiversity is widened in scope and deeply values and appreciates these differences as strengths, not limitations or deficiencies. Neurodiversity was covered in several studies in this review. Individual differences and uniqueness were accounted for in six studies (7, 14, 19, 26, 30, 34, 35). Coverage of psychiatric co-morbidities were assessed in two studies (6, 34). Co-morbid sensory complications were reviewed in content in two studies (6, 7). Autism as an inherent part of identity was examined in one study (7). Empower in embracing neurodiversity was covered in one study (8). Lastly, one study described content on asset and superpower for the autistic population (15).

### 3.11. Medical model

With respect to the medical model approach, only one study presented content on autism as a disorder or defect (6).

### 3.12. Autism awareness and advocacy

Autism Awareness and Advocacy took many forms in content covered across published studies. World Autism Awareness Day was accounted for in three studies (19, 21, 23). Autism Speaks Advocacy Organization was referenced in content described in two studies (22, 23). The Action Summit on Autism was featured in one study (22). Representation of the color blue was depicted in two studies (19, 23). Books on autism were covered in two studies (8, 33). Media coverage of autism was accounted for in one study (8). Celebrity endorsements were assessed in one study as well (8). Coverage of fundraising was accounted for in two studies (8, 33). One study uncovered content on healthcare providers participating in interviews (19). Community engagement was accounted for in three studies (23, 30, 31). Autism-related research was featured in three studies (30, 31, 35). Jewelry and tattoos were both reviewed in one study (33). Lastly, trainings, workshops, and conferences for knowledge transfer and dissemination were examined in one study (30).

### 3.13. Recreation

Recreation coverage involved toys and gifts for children with autism (12), sports and recreational activities (20), and play groups (33).

### 3.14. Misinformation

Across misinformation, three studies identified content on immunizations and vaccinations as contributing factors to the onset and exacerbation of autism (7, 8, 12). MMR vaccine, scarcity of vitamins, environmental pollution, toxicity, and scarcity of amino acids were content covered in one study (12). Two studies assessed content on gluten-free diet as a cure for autism (12, 34). Lastly, one study depicted content on asymmetrical brain injuries attributed to autism (16).

## 4. Discussion

This scoping review involved the review of published studies on content analyses across a range of considerations surrounding autism across social media platforms. The premise of this review was to present the existing state of research and practice in this domain as the basis to inform future work in utilizing the social media landscape as a health education and communication medium on clinical and practical recommendations and considerations in understanding and navigating autism. In addition, although the published studies in this review examined content posted by individuals with autism, another focus of this review was to examine published content on autism posted by a diversity of sources across social media platforms, not solely among the neurodivergent community with autism on social media.

Each published study examined content reporting autism content. This review consolidated the key takeaways from the constellation of this literature as the basis to clearly identify the existing state of knowledge, research and practice on autism across promising health communication mediums (i.e., social medial platforms). Across this review, there were a total of 32 sources that covered content on autism across YouTube, Twitter, and Facebook. There was significant dispersion in the coverage of topics, likely attributed to variations in the key words and phrases as well as methodologies across studies. Further, majority of the studies utilized terms that were more research-oriented and thereby less likely to be searched by consumers/non-professionals.

These findings present an incomplete understanding of the terminology that will capture the most widely viewed, engaging, and helpful content for viewers with autism or who have someone in their networks with autism (e.g., caregiver of an autistic child). Most studies involved content analyses of the widely covered content on autism but some of them had a more delimited scope. In fact, several of the studies solely examined content in specific formats (e.g., home videos) as well as ones posted by specific sources (e.g., caregivers of autistic children, autism bloggers). In addition, there were variations in sample sizes of videos, tweets, messages and comments analyzed among studies likely related to the different methodologies across these studies. Nevertheless, findings from this review help elucidate the existing state of knowledge and practice surrounding autism presented on YouTube, Twitter, and Facebook as rising communication mediums in our digital era. Furthermore, these findings inform future directions for research and practice on the utilization of social media to disseminate up-to-date, resourceful content to viewers closely or distally affected by autism.

### 4.1. Diversity, equity, and inclusion implications

Eight studies covered content across YouTube and Twitter on diversity, equity, and inclusion (DEI) considerations (7, 9, 11, 19, 23, 34, 35). It is possible that minimal coverage of these considerations could be based on the limitations of the search strategies across the studies in this review. None of the studies accounted for DEI language in their search strategies which could further hinder uncovering content that promote equity and inclusivity. In addition, there were nine studies that accounted for neurodiversity considerations and embraced individuals with autism as different without any label attached (6–8, 14, 15, 19, 26, 34, 35). Future work could account for ways to find the most up-to-date and engaging content on these considerations in supporting individuals with autism as well as their networks from all walks of life.

### 4.2. Autism-related policies and legislation

Of note, none of the studies covered content across videos pertaining to policy measures in place to promote protection, equity, accessibility, and inclusivity for individuals with autism. Policy measures are crucial in casting a wider net of support for these individuals across community, national, and global contexts. There are several community and national policies in place to support individuals with neurobehavioral and developmental disabilities. National policies include the Developmental Disabilities Act and further legislation that followed since its inception. In addition, the Rehabilitation Act of 1973 requires that all public schools across the USA provide free and accessible public education (FAPE) to students with disabilities. This federal legislation extends to all states in the U.S. In Brazil, a federal law recognizes autism as a disability for legal purposes as the basis to secure social benefits for individuals with autism (38). England enacted legislation for adults and children that heightened protection of rights for both of these populations with autism as well as increased access to services which could meet the range of their medical, developmental, educational, financial, and additional needs and accommodations (39). Scotland and Ireland both have policies in place to assure access to equitable educational opportunities for individuals with autism (39). Coverage of these policies among content on social media could heighten their awareness and visibility in creating an inclusive and equitable space for individuals with autism.

### 4.3. Patient education considerations

In this digital era, online support networks are ubiquitous. In fact, the development of these groups may yield more connection among participants across the world than ever possible in-person (40). The information shared across these electronic communication mediums could be accessed much more readily by patients with autism and their support networks than through a health systems approach. There are a range of healthcare providers involved in the multidisciplinary care of patients with autism that include developmental pediatricians, speech language pathologists, psychologists, neurologists, psychiatrists, and much more. Resources provided by healthcare providers are oftentimes more informational in nature and can include suggestions on programs for global development, additional therapies, and medication management. Healthcare providers are in a prime position to integrate more resources that could benefit their patients in navigating the chronicity of autism. By learning more about these online support networks and assessing for risk and degree of misinformation, healthcare providers could uncover one or more of them that present accurate information and in turn could share content about these networks as adjunct resources for their patients. In addition, healthcare providers could also find ways to increase their presence on social media platforms as the basis to mitigate misinformation and disinformation surrounding knowledge of autism and recommendations in care and management. Further in this context, healthcare providers are also in an optimal position to recommend when to seek healthcare based on the nature of concerns and experiences shared by individuals with autism and their support networks on these virtual spaces.

### 4.4. Recommendations to offset misinformation

It is crucial to account for the monetization of social media platforms in the credibility of content delivered across these spaces. YouTube, Twitter, and Facebook are no exception and given the wealth of publicly available content that are posted and continued to be posted, it is no surprise that misleading, misguided information will be included in content by predatory influencers. For this reason, finding ways to mitigate the impact of misinformation and disinformation is of paramount importance in the health education landscape. One strategy could involve integrating the health systems approach with social media in increasing the presence of credible sources on prominent social media platforms which in turn yields promise in having a downstream impact on optimizing care and quality of life for more viewers with autism as well as their support networks. Content that involves expeditious curative approaches to autism are more likely disseminating misinformation given that there is no cure for autism. It follows that the presence of healthcare providers could present scientific and up-to-date content as part of the comments and messages or as separate videos to offset the dissemination and impact of this misinformation. Of note, one study uncovered dangerous, harmful strategies across videos that promised cures (11). Increasing the visibility of positive influencers without any monetary stake on social media could be another promising approach in strengthening the credibility and accuracy of content across YouTube, Twitter, and Facebook.

### 4.5. Campaigns and additional awareness strategies

Only four of the studies covered content on prominent autism-related campaigns and additional measures across the world on promoting advocacy and awareness of Autism (19, 21–23). For example, the World Autism Awareness Day in April of each year was established by the United Nations to heighten knowledge and awareness on autism and mobilize advocacy by organizations and consumers on embracing its diversity through a cascade of educational efforts across the globe (41). Finding ways to make content across these campaigns and awareness measures more engaging and appealing to viewers could increase their visibility not just on one social media platform but across several as content on one social media platform could also be posted on others. In addition, global organizations could take a more active role in posting videos about the nationally recognized day leading up to the actual day which in turn could heighten more knowledge, awareness, and advocacy efforts in reducing inequities for this patient population. Further, campaigns oftentimes feature testimonials. Given that there were multiple videos across studies on YouTube that presented testimonials which garnered large views, it could help to engage consumers in collaborating with campaign developers to create content that will not only increase the popularity and visibility of the campaign but also continue to impart narratives as an appealing educational tool in understanding a range intricacies and complexities surrounding autism from the ones living through it.

### 4.6. Implications for organizational guidelines

Notably, majority of the videos, messages, comments, and hashtags across studies were posted by consumers. Guidelines developed by the American Academy of Pediatrics, Centers for Disease Control and Prevention, American Psychiatric Association, and other entities to support early diagnosis and treatment of autism were not covered among the content on each examined social media platform. Many of the videos spanned the past 16 years from the time of YouTube's inception. Furthermore, cross-sectional samples of tweets and online groups on Facebook were obtained from the past decade. It follows that these organizations may not be doing enough to support individuals with autism and their networks as their content is not among the widely viewed videos on YouTube and further also did not appear amidst an assortment of search strategies on yielding information across multiple dimensions of autism on YouTube, Twitter, and Facebook. Similar to campaigns, it is possible that creating collaborative spaces for consumers to help these organizations in developing autism-related content could spark up more engagement and in turn increase viewership.

### 4.7. Content on medications and therapies

There were several studies yielding content on social media that delivered communication on medication advice from caregivers of autistic children. Also among this content, recommendations for different kinds of speech language therapies, applied behavior analysis, and more interventions were presented by caregivers of children, not healthcare providers. These findings suggest that consumers are more likely to engage with their fellow consumers in learning more about the range of medical treatments available in the chronic care of autism for their children. There could also be more trust in the information shared given that consumers are learning information directly from another consumer with a shared lived experience. This connectivity could potentially outweigh guidance provided by healthcare providers who do not share this lived experience. It follows that healthcare providers could learn from consumers in strengthening their practice, empathy, and provision of resources for patients with autism and their families.

### 4.8. Media portrayal of autism

Findings from this review uncovered a wider range of autism-related content on social media which is different from findings across published studies on traditional media representation of autism. Across multiple published studies that reviewed portrayal of autism across news media sources, content pertained to stigma, social attitudes, quality of portrayal with respect to meeting DSM-V criteria for autism, and stereotypes surrounding autism (42–48). Across published studies on films, television series, fiction books, and online shows, representation of autism was sometimes depicted in characters with autism. These studies also yielded autism-related content on stereotypes (e.g., intellectual geniuses, inspiring heroes, characters with special abilities, individuals facing emotional instability), social attitudes (e.g., stigma, fascination, empathy, and acceptance, perspectives on causality pertaining to internal or external triggers for autism, prevention, and stability), quality of portrayal with respect to meeting partial or complete DSM-V criteria for autism, as well as benefits and downsides to society (e.g., increased social awareness of autism neurotypes) (49–60). Future work could also account for the totality of media coverage across both traditional and nontraditional sources of media in presenting consistent information across these communication mediums on more expansive domains, dimensions and determinants surrounding autism to guide interventions that promote awareness, advocacy, inclusivity, and equity for autistic individuals.

### 4.9. Neurodivergence representation on TikTok

As TikTok continues to trend as a prevalent social media platform, it is crucial for future work in this domain to assess messaging, active discussions, posts, and other methods of communication across this social media platform on autism. Given the recent emergence of TikTok, there is only one published study assessing autism-related content covered on TikTok to date (61). Future studies could critically examine this content and further explore whether the bearers of information, misinformation or disinformation are connected to credible or lay sources along with how much engagement this content attracts among viewers. TikTok is increasingly trending as a preferred social media platform for the neurodivergent community with increased visibility of users with ADHD and autism (61, 62). In addition, TikTok is trending in popularity as a social network among teenagers (63). Furthermore, TikTok is also rising among adults as a preferred news media medium (64). Taking everything into consideration, this platform offers another opportunity to engage with users and influencers to mitigate misinformation and disinformation alongside promoting the credible knowledge transfer and dissemination on autism for these large segments of both the U.S. national and global populations.

### 4.10. Implications for health equity and access to resources

Most studies did not involve content with a balanced representation of individuals with autism from different racial, ethnic, age, and socioeconomic groups. Individuals residing in resource limited settings are more likely to be of lower socioeconomic status which in turn could significantly delimit access to resources that could be prevalent in more resource rich settings. In addition, marginalized populations may be more likely to be uninsured and underinsured which may also present a barrier to accessing timely and needed care in navigating the specialized and complex needs surrounding autism. Given that access to technology in our digital era is prevalent (36), social media presents an open marketplace of knowledge, strategies, and additional considerations for individuals who are uninsured and underinsured. Until there is increased access to resources for these individuals, we could direct them and their networks to the availability of autism-related resources on social media as a bridge in reducing health inequities stemming from resource limitations which furthermore could increase cost efficiency across the healthcare system. In addition, given the chronicity of autism, it is crucial for future clinical practice to view autism as a chronic illness that warrants care and resource provision on a continuum.

### 4.11. Recommendations for further research

As autism continues to be a common neurotype across the globe, it is crucial to continue further work in understanding a range of dimensions as the basis to support patients with autism across developmental stages. In addition, it is also important to note that awareness of autism as a diverse lived experience is growing around the globe, as is the ability of individuals to self-diagnose or seek formal diagnosis secondary to better knowledge of the condition. In turn, this is reshaping the medical community's understanding of autism as well as people's lived experience of it. Health education in this domain is timely and takes many forms. As noted across the studies in this review, social media is a promising virtual space and communication medium for the dissemination of knowledge and support to these patients and their networks.

Future studies could examine videos, tweets, comments, and messages on autism in different languages. In this review, only one study explored videos in Spanish. Examining more content prevalent in different languages will increase access to videos that could be useful for viewers from different countries where autism incidence and prevalence rates are high as well.

In addition across most studies on YouTube, view count was the sole primary metric for assessing viewer engagement. Among all studies in this review, only four studies thoroughly examined comments posted by viewers (12, 16, 32, 35). Future research could involve conducting a thematic analysis of comments to critically assess them for viewer engagement, acceptability of content as well as knowledge, attitudes, and perspectives of viewers.

Several of the studies mentioned alternative complementary practices (e.g., animal, art, and herbal therapies) which are not necessarily in line with existing guidelines provided the American Academy of Pediatrics, the CDC, and other notable organizations in the diagnosis and intervention of autism. Future research could involve conducting longitudinal and prospective studies to assess the efficacy of these practices as the basis for consideration of integrating them into evidence-based practices for early intervention among children with autism.

Search strategies were different across the studies in this review. Further, goals of several of the studies were to capture content posted by families of children with autism. Future work could account for drawing on the strengths of some of the search strategies and building off the limitations of others in creating a more robust search strategy that will yield the most helpful content in supporting viewers with autism and their networks.

Majority of the studies focused on content pertaining to children with autism. Many children live into adulthood and face newer challenges in navigating their lives as they grow older with autism. It follows that another direction for future research could explore videos, messages, and hashtags related to adults with autism presenting their narratives and perspectives inclusive of their experiences, daily challenges, therapies, and more considerations as the basis to increase knowledge and support fellow adults with autism and their networks.

Of note, only one study truly examined the nature of content with respect to whether they supported the medical model or the neurodiversity paradigm in understanding and navigating autism. Oftentimes, the coverage of topics on social media matches with trends around the world. One of these trends is to embrace diversity across all walks of life. Neurodiversity supports the utilization of terminology in line with individual and group differences rather than defects or disorders that align more with the medical model. Uncovering content that truly capture elements of neurodiversity could be another direction for further research. Specifically exploring the qualitative nature of this content, educational value, and degree of support they can offer to viewers with autism, their networks, and treating providers could yield promise in mobilizing worldwide advocacy, awareness, and inclusivity of autism.

Lastly, the current review addresses content covered on autism across the most prominent social media platforms and includes organizational, governmental, consumer and other kinds of sources presenting information in several different formats, for both the generalized global lay population as well as specialized populations inclusive of the autistic community worldwide. One consolidated review specifically appraises literature on the user experience on social media for autistic individuals (65). Future work could also continue to examine social media utilization and engagement among individuals with autism in knowledge transfer and dissemination and building a sense of community.

### 4.12. Limitations

There were several limitations in this review. First, given the scoping narrative and descriptive nature of this review, it ultimately did not involve conducting rigorous composite statistical analyses to critically examine study biases found in a systematic review. Furthermore, all sources examined were in English, thereby precluding any sources in different languages which could be relevant to understanding the landscape of autism covered on social media. In addition, given the potential bias in sample size and selection methods as well as variations in the search strategies across studies, it is possible that widely viewed and accessed content may not have been integrated in one or more of the samples of videos, tweets, comments, and messages. It is also important to note that all studies involved cross-sectional study designs which offered a snapshot of content related to autism. Given the ever-changing landscape of social media with the evolution of videos, messages, hashtags and other forms of posts, it is possible that the coverage of content in the present time could be substantially different among the widely viewed posts. In addition, the number of views and likes that these posts generate are constantly changing. Successive sampling of posts through continued content analyses and narrative inquiries could further examine any changes in the coverage of autism across YouTube, Twitter, and Facebook. Lastly, other social media platforms (e.g., Instagram, TikTok) were not included in this review given the dearth of findings on autism-related content covered across them.

## 5. Conclusion

Autism continues to remain among the most widely prevalent neurobehavioral disorders worldwide. Early intervention through increased access to timely resources and services could yield promise in offsetting adverse health and developmental consequences for individuals with autism and their networks. In the context of our digital era, social media has increased its visibility as a communication medium for health education extending to autism. Given the ubiquitous nature of social media, there is increased efficiency in accessing content that could yield support and knowledge to viewers with autism and their networks. Accounting for the increased benefits of knowledge transmission but also the downfalls attributed to misinformation is crucial given the monetization of social media platforms and subsequent predatory influencers that could distort accuracy of content. It is also imperative to focus on policy, campaign and equity considerations in mitigating disparities for the global autistic population. Future work could extend on these studies to uncover more content that could be integrated into the care of patients with autism.

## Data Availability

The original contributions presented in the study are included in the article/Supplementary Material, further inquiries can be directed to the corresponding author.

## References

[1] Centers for Disease Control and Prevention. *Signs and symptoms of autism spectrum disorder*. (2022). Available at: https://www.cdc.gov/ncbddd/autism/signs.html

[2] Centers for Disease Control and Prevention. Prevalence of autism spectrum disorder among children aged 8 years—autism and developmental disabilities monitoring network, 11 sites, United States 2014. Morb Mort Weekly Rep Surv Summ. (2018) 67:1–23. 10.15585/mmwr.ss6706a1 PMC5919599 29701730

[3] Alpert JS. Autism: a spectrum disorder. Am J Med. (2021) 134:701–2. 10.1016/j.amjmed.2020.10.022 33181106

[4] American Psychiatric Association. Diagnostic and statistical manual of mental disorders. 5th ed. Virginia: American Psychiatric Publishing (2013).

[5] Pews Research Center. *Social media use in 2021*. (2021). Available at: https://www.pewresearch.org/internet/2021/04/07/social-media-use-in-2021/

[6] Angulo-Jimenez H DeThorne L. Narratives about autism: an analysis of YouTube videos by individuals who self-identify as autistic. Am J Speech Lang Pathol. (2019) 28:569–90. 10.1044/2018_AJSLP-18-0045 30995116

[7] Kollia B Kamowski-Shakibai MT Basch CH Clark A. Sources and content of popular online videos about autism spectrum disorders. Health Promot Perspect. (2017) 7:238–44. 10.15171/hpp.2017.41 29085802 PMC5647360

[8] Bellon-Harn ML Manchaiah V Morris LR. A cross-sectional descriptive analysis of portrayal of autism spectrum disorders in YouTube videos: a short report. Autism. (2020) 24:263–8. 10.1177/1362361319864222 31303030

[9] Bellon-Harn ML Ni J Manchaiah V. Twitter usage about autism spectrum disorder. Autism. (2020) 24:1805–16. 10.1177/1362361320923173 32508126

[10] Fusaro VA Daniels J Duda M DeLuca TF D’Angelo O Tamburello J The potential of accelerating early detection of autism through content analysis of YouTube videos. PLoS One. (2014) 9:e93533. 10.1371/journal.pone.0093533 24740236 PMC3989176

[11] Lacruz-Perez I Sanz-Cervera P Pastor-Cerezuela G Gomez-Mari I Tarraga-Minguez R. Is it possible to educate, intervene or “cure” autism Spectrum disorder? A content analysis of YouTube videos. Int J Environ Res Public Health. (2021) 18:2350. 10.3390/ijerph18052350 33670846 PMC7967735

[12] Azer SA Bokhari RA AlSaleh GS Alabdulaaly MM Ateeq KI Guerrero APS Experience of parents of children with autism on YouTube: are there educationally useful videos? Inform Health Soc Care. (2018) 43:219–33. 10.1080/17538157.2018.1431238 29461878

[13] Lloyd S Osborne LA Reed P. Personal experiences disclosed by parents of children with autism spectrum disorder: a YouTube analysis. Res Autism Spectr Disord*.* (2019) 64: 13–22. 10.1016/j.rasd.2019.03.009

[14] Freitas BMS Gaudenzi P. “We, mothers of autistic people”: between knowledge of the experience and collective memories in videos on YouTube. Cien Saude Colet. (2022) 27:1595–2022. 10.1590/1413-81232022274.07212021 35475838

[15] Skafle I Gabarron E Dechsling A Nordahl-Hansen A. Online attitudes and information-seeking behavior on autism, asperger syndrome, and Greta Thunberg. Int J Environ Res Public Health. (2021) 18:4981. 10.3390/ijerph18094981 34067114 PMC8124294

[16] Gabarron E Dechsling A Skafle I Nordahl-Hansen A. Discussions of asperger syndrome on social media: content and sentiment analysis on Twitter. JMIR Form Res. (2022) 6:1–6. 10.2196/32752 PMC8938830 35254265

[17] Gauld C Maquet J Micoulaud-Franchi JA Dumas G. Popular and scientific discourse on autism: representational cross-cultural analysis of epistemic communities to inform policy and practice. J Med Internet Res. (2022) 24:e32912. 10.2196/32912 35704359 PMC9244652

[18] Corti L Zanetti M Tricella G Bonati M. Social media analysis of twitter tweets related to ASD in 2019–2020, with particular attention to COVID-19: topic modelling and sentiment analysis. J Big Data. (2022) 9:1–7. 10.1186/s40537-022-00666-4 PMC9702597 36465137

[19] Sani-Bozkurt S. A comparative examination of social perception, network structure, important nodes, and discourses regarding ASD awareness over online networks: a social network and content analysis. J Educ Technol Online Learn. (2021) 4:293–309. 10.31681/jetol.924084

[20] Deriss MJ. Review of topics related to autism spectrum disorder on twitter. Netw Model Anal Health Inform Bioinforma. (2019) 8:1–6. 10.1007/s13721-019-0195-3

[21] Ahmed W Bath PA Sbaff L Demartini G. Measuring the effect of public health campaigns on Twitter: the case of world autism awareness day. In: Chowdhury G. McLeod J. Gillet V. Willett P. (eds) Transforming digital worlds. iConference 2018. Lecture notes in computer science, vol. 10766. Cham: Springer (2018). 10.1007/978-3-319-78105-1_2

[22] Beykikhoshk A Arandjelović O Phung D Venkatesh S Caelli T. Data-mining Twitter and the autism spectrum disorder: a pilot study. 2014 IEEE/ACM international conference on advances in social networks analysis and mining (ASONAM 2014) (2014). p. 349–56. 10.1109/ASONAM.2014.6921609

[23] Karusala N. Kumar N Arriaga R. #Autism: Twitter as a Lens to explore differences in autism awareness in India and the United States. In tenth international conference on information and communication technologies and development (ICTD ‘19), January 4–7, 2019, Ahmedabad, India. New York, NY, USA: ACM (2019). p. 5. 10.1145/3287098.3287137

[24] Beykikhoshk A Arandjelović O Phung D Venkatesh S. Overcoming data scarcity of Twitter: using tweets as bootstrap with application to autism-related topic content analysis. 2015 IEEE/ACM international conference on advances in social networks analysis and mining (ASONAM) (2015). p. 1354–61. 10.1145/2808797.2808908

[25] Saha A Agarwal N. Insight into social support of autism blogger community in microblogging platform. 2015 AAAI spring symposium series; 2015 Mar 12.

[26] Saha A Agarwal N. Modeling social support in autism community on social media. Netw Model Anal Health Inform Bioinforma*.* (2016) 5:8. 10.1007/s13721-016-0115-8

[27] Saha A Agarwal N. Demonstrating social support from autism bloggers community on Twitter. In: Proceedings of the 2015 IEEE/ACM international conference on advances in social networks analysis and mining 2015; 2015 Aug 25; pp. 1053–6.

[28] Beykikhoshk A Arandjelović O Phung D Venkatesh S Caelli T. Using Twitter to learn about the autism community. Soc Netw Anal Min. (2015) 5:22. 10.1007/s13278-015-0261-5

[29] Ward DM Dill-Shackleford KE Mazurek MO. Social media use and happiness in adults with autism spectrum disorder. Cyberpsychol Behav Soc Netw. (2018) 21:205–9. 10.1089/cyber.2017.0331 29485900

[30] Zhao Y Zhang J Wu M. Finding users’ voice on social media: an investigation of online support groups for autism-affected users on Facebook. Int J Environ Res Public Health. (2019) 16:4804. 10.3390/ijerph16234804 31795451 PMC6926495

[31] Mohd Roffeei SH Abdullah N Basar SK. Seeking social support on Facebook for children with autism spectrum disorders (ASDs). Int J Med Inform. (2015) 84:375–85. 10.1016/j.ijmedinf.2015.01.015 25701266

[32] Bail CA. Emotional feedback and the viral spread of social media messages about autism spectrum disorders. Am J Public Health. (2016) 106:1173–80. 10.2105/AJPH.2016.303181 27196641 PMC4984751

[33] Abel S Machin T Brownlow C. Support, socialise and advocate: an exploration of the stated purposes of Facebook autism groups. Res Autism Spectr Disord. (2019) 61:10–21. 10.1016/j.rasd.2019.01.009

[34] Antunes D Dhoest A. Autism and social media: the case of Brazil. Observatorio (OBS*). (2018) 12:1–12. 10.15847/obsOBS12420181298

[35] Bakombo S Ewalefo P Konkle ATM. The influence of social media on the perception of autism Spectrum disorders: content analysis of public discourse on YouTube videos. Int J Environ Res Public Health. (2023) 20:3246. 10.3390/ijerph20043246 36833941 PMC9961260

[36] Cortes Cavalcante J Faria Sales M Sousa Junior RR Souto DO Vale Gonçalves R Camargos ACR Analysis of the Brazilian-Portuguese content on autism spectrum disorder available on YouTube videos. Phys Occup Ther Pediatr. (2023) 17:1–15. 10.1080/01942638.2023.2199843 37069791

[37] Goksel P Oban V Dikec G Usta MB. Qualitative and artificial intelligence-based sentiment analysis of Turkish twitter messages related to autism spectrum disorders. Cureus. (2023) 15:e38446. 10.7759/cureus.38446 37143854 PMC10153655

[38] Rios C Costa Andrada B. The changing face of autism in Brazil. Cult Med Psychiatry. (2015) 39:213–34. 10.1007/s11013-015-9448-5 25842350

[39] Roleska M Roman-Urrestarazu A Griffiths S Ruigrok ANV Holt R van Kessel R Autism and the right to education in the EU: policy mapping and scoping review of the United Kingdom, France, Poland and Spain. PLoS One. (2018) 13(8):e0202336. 10.1371/journal.pone.0202336 30161146 PMC6116926

[40] Coulson NS Buchanan H. The role of online support groups in helping individuals affected by HIV and AIDS: scoping review of the literature. J Med Internet Res. (2022) 24(7):e27648. 10.2196/27648 35881456 PMC9364165

[41] United Nations. *World autism awareness day*. (2022). Available at: https://www.un.org/en/observances/autism-day

[42] Jones SC Harwood V. Representations of autism in Australian print media. Disabil Soc. (2009) 24(1):5–18. 10.1080/09687590802535345

[43] Holton AE Farrell LC Fudge JL. A threatening space? Stigmatization and the framing of autism in the news. Commun Stud. (2014) 65(2):189–207. 10.1080/10510974.2013.855642

[44] Thys E Struyven CI Danckaerts M De Hert M. The stigmatising of schizophrenia and autism in the flemish daily papers. Tijdschr Psychiatr. (2014) 56(6):365–74. Available at: www.ncbi.nlm.nih.gov/pubmed/24953510 24953510

[45] Bie B Tang L. Representation of autism in leading newspapers in China: a content analysis. Health Commun. (2015) 30(9):884–93. 10.1080/10410236.2014.889063 25074820

[46] Tang L Bie B. The stigma of autism in China: an analysis of newspaper portrayals of autism between 2003 and 2012. Health Commun. (2016) 31(4):445–52. 10.1080/10410236.2014.965381 26398334

[47] Huws JC Jones RSP. Missing voices: representations of autism in British newspapers, 1999–2008. Br J Learn Disabil. (2011) 39(2):98–104. 10.1111/j.1468-3156.2010.00624.x

[48] Baeyens D Moniquet A Danckaerts M van der Oord S. Vergelijkend onderzoek naar structureel stigma bij ADHD en autismespectrumstoornis in de vlaamse dagbladen. Tijdschr Psychiatr (2017) 59(5):269–77. Available at: www.narcis.nl/publication/RecordID/oai:dare.uva.nl: publications%2Fa029da2a-091e-4ebd-b449-0405ec8e50b7 28593620

[49] Draaisma D. Stereotypes of autism. Philos Trans R Soc B. (2009) 364(1522):1475–80. 10.1098/rstb.2008.0324 PMC2677582 19528033

[50] Pourre F Aubert E Andanson J Raynaud J. Le syndrome d’Asperger dans les o’uvres de fiction actuelles. L’Ence’Phale. (2012) 38(6):460–66. 10.1016/j.encep.2011.12.009 23200611

[51] Belcher C Maich K. Autism spectrum disorders in popular media: storied reflections of societal views. Brock Educ. (2014) 23(2):97–115. 10.26522/brocked.v23i2.311

[52] Garner A Jones S Harwood V. Authentic representations or stereotyped “outliers”: using the CARS2 to assess film portrayals of autism spectrum disorders. Int J Cult Ment Health. (2015) 8(4):414–25. 10.1080/17542863.2015.1041993

[53] Lemoine L Mietkiewicz M Schneider B. Autism told to children: can children’s literature be used to promote awareness? Enfance. (2016) 2(2):231–45. 10.4074/S0013754516002056

[54] Lugo N Melon ME Castillo MC. La representaci on del autismo en las narrativas de fanfiction.net: los espacios de afinidad como oportunidad para la negociacion de sentido. Palabra Clave Revista de Comunicacion. (2017) 20(4):948–78. 10.5294/pacla.2017.20.4.5

[55] Nordahl-Hansen A. Atypical: a typical portrayal of autism? Lancet Psychiatry. (2017) 4(11):837–38. 10.1016/S2215-0366(17)30397-8 29115250

[56] Nordahl-Hansen A Øien RA Fletcher-Watson S. Pros and cons of character portrayals of autism on TV and film. J Autism Dev Disord. (2017) 48(2):635–36. 10.1007/s10803-017-3390-z 29170934

[57] Nordahl-Hansen A Tøndevold M Fletcher-Watson S. Mental health on screen: a DSM-5 dissection of portrayals of autism spectrum disorders in film and TV. Psychiatry Res. (2017) 262:351–53. 10.1016/j.psychres.2017.08.050 28843629

[58] Black R Alexander J Chen V Duarte J. Representations of autism in online Harry Potter fanfiction. J Lit Res. (2019) 51(1):30–51. 10.1177/1086296X18820659

[59] Moore A. He’s not rain man: representations of the sentimental savant in ABC’s the good doctor. J Pop Telev. (2019) 7(3):299–316. 10.1386/jptv_00003_1

[60] Tharian PR Henderson S Wathanasin N Hayden N Chester V Tromans S. Characters with autism spectrum disorder in fiction: where are the women and girls? Adv Autism. (2019) 5(1):50–63. 10.1108/AIA-09-2018-0037

[61] Gilmore D Radford D Haas MK Shields M Bishop L Hand B. Building community and identity online: a content analysis of highly viewed# autism TikTok videos. Autism Adulthood. (2023). 10.1089/aut.2023.0019 PMC10902280 38435322

[62] McDermott V. “Tell me something you didn’t know was neurodivergence-related until recently. I’ll start”: TikTok as a public sphere for destigmatizing neurodivergence. In: Nerren JB, editor. Rethinking perception and centering the voices of unique individuals: Reframing autism inclusion in praxis. San Bernardino: IGI Global. (2022). pp. 127–47.

[63] Vogels EA Gelles-Watnick R Massarat N. *Teens, social media and technology 2022*. (2022). Available at: https://www.pewresearch.org/internet/2022/08/10/teens-social-media-and-technology-2022/ (Accessed on July 18, 2023).

[64] Matsa KE. *More Americans are getting news on TikTok, bucking the trend on other social media sites*. (2022). Available at: https://www.pewresearch.org/short-reads/2022/10/21/more-americans-are-getting-news-on-tiktok-bucking-the-trend-on-other-social-media-sites/ (Accessed on July 18, 2023).

[65] Hudson NA Linnane JM Rayner-Smith K. Autism and social media: a systematic review of the user experience. Adv Autism. (2023) 9(3):201–16. 10.1108/AIA-01-2023-0001

